# Gene expression profiling in susceptible interaction of grapevine with its fungal pathogen Eutypa lata: Extending MapMan ontology for grapevine

**DOI:** 10.1186/1471-2229-9-104

**Published:** 2009-08-05

**Authors:** Ana Rotter, Céline Camps, Marc Lohse, Christian Kappel, Stefania Pilati, Matjaž Hren, Mark Stitt, Pierre Coutos-Thévenot, Claudio Moser, Björn Usadel, Serge Delrot, Kristina Gruden

**Affiliations:** 1 National Institute of Biology, Department of Biotechnology and Systems Biology, Večna pot 111, 1000 Ljubljana, Slovenia; 2 Institute of Vine and Wine Sciences (ISVV), University Victor Segalen Bordeaux II, Unite Mixte de Recherches Ecophysiology and Grape Functional Genomics, INRA, 71 Avenue Edouard, Bourlaux 33883, BP 81, Villenave d'Ornon, France; 3 Max Planck Institute of Molecular Plant Physiology Am Mühlenberg 1, 14476 Golm, Germany; 4 Department of Genetics and Molecular Biology, IASMA Research Center, Via E. Mach 1, 38010 S, Michele a/Adige (TN), Italy; 5 Laboratoire de Physiologie et Biochimie Végétales, UMR CNRS 6161, Université de Poitiers, Bâtiment Botanique, 40 Avenue du Recteur Pineau, 86022 Poitiers Cedex, France

## Abstract

**Background:**

Whole genome transcriptomics analysis is a very powerful approach because it gives an overview of the activity of genes in certain cells or tissue types. However, biological interpretation of such results can be rather tedious. MapMan is a software tool that displays large datasets (e.g. gene expression data) onto diagrams of metabolic pathways or other processes and thus enables easier interpretation of results. The grapevine (*Vitis vinifera*) genome sequence has recently become available bringing a new dimension into associated research. Two microarray platforms were designed based on the TIGR Gene Index database and used in several physiological studies.

**Results:**

To enable easy and effective visualization of those and further experiments, annotation of *Vitis vinifera *Gene Index (VvGI version 5) to MapMan ontology was set up. Due to specificities of grape physiology, we have created new pictorial representations focusing on three selected pathways: carotenoid pathway, terpenoid pathway and phenylpropanoid pathway, the products of these pathways being important for wine aroma, flavour and colour, as well as plant defence against pathogens. This new tool was validated on Affymetrix microarrays data obtained during berry ripening and it allowed the discovery of new aspects in process regulation. We here also present results on transcriptional profiling of grape plantlets after exposal to the fungal pathogen *Eutypa lata *using Operon microarrays including visualization of results with MapMan. The data show that the genes induced in infected plants, encode pathogenesis related proteins and enzymes of the flavonoid metabolism, which are well known as being responsive to fungal infection.

**Conclusion:**

The extension of MapMan ontology to grapevine together with the newly constructed pictorial representations for carotenoid, terpenoid and phenylpropanoid metabolism provide an alternative approach to the analysis of grapevine gene expression experiments performed with Affymetrix or Operon microarrays. MapMan was first validated on an already published dataset and later used to obtain an overview of transcriptional changes in a susceptible grapevine – *Eutypa lata *interaction at the time of symptoms development, where we showed that the responsive genes belong to families known to be involved in the plant defence towards fungal infection (PR-proteins, enzymes of the phenylpropanoid pathway).

## Background

Several tools are now available to describe plants metabolic pathways (see [1] for a recent review on Reactome), but they are usually restricted to specific parts of the metabolism. However, it is often interesting to get a quick and complete overview of the whole data set, especially at the start of the analysis. Furthermore, many tools are only offered as online-tools, thus the user has to rely on the availability of a fast internet connection at the time of the analysis. To overcome these problems, MapMan [2] introduced an ontology which removes redundancies, and displays metabolic maps including many processes at once, thus immediately highlighting important pathways. Later on, statistical tools [3] were added to this software package to get an unbiased overview of changed pathways or processes. The ontology was originally built for the model species *Arabidopsis thaliana*, and furthermore extended to cover also maize [4], Medicago [5], tomato [6] and potato [7]. Briefly, MapMan ontology consists of a set of 34 hierarchical BINs (Table 1), constructed around central metabolism, as well as other categories (e.g. stress, cell etc.). Original BIN assignments were based on publicly available gene annotation within TIGR (The Institute for Genomic Research), adopting a process alternating between automatic recruitment and manual correction [2]. BINs can be furthermore split into hundreds of subBINs.

**Table 1:** MapMan BIN structure and number of manual corrections made for each BIN

**BIN**	**BIN name**	**No. of clones in the BIN**	**No. of corrected clones**	% of **corrected clones**
1	photosynthesis	494	23	4.6
2	major CHO metabolism	165	8	4.8
3	minor CHO metabolism	162	13	8
4	glycolysis	123	9	7.3
5	fermentation	52	0	0
6	gluconeogenesis/glyoxylate cycle	22	2	9
7	oxidative pentose phosphate pathway	42	1	2.4
8	TCA cycle/org. acid transformations	123	8	6.5
9	mitochondrial electron transport/ATP synthesis	156	4	2.6
10	cell wall	595	4	0.7
11	lipid metabolism	495	27	5.9
12	nitrogen metabolism	59	4	6.8
13	amino acid metabolism	459	17	3.7
14	sulphur assimilation	15	0	0
15	metal handling	142	14	9.9
16	secondary metabolism	543	92	16.9
17	hormone metabolism	502	29	5.8
18	cofactor and vitamin synthesis	45	3	6.7
19	tetrapyrrole synthesis	56	14	25
20	stress	948	456	48.1
21	redox	282	15	5.3
22	polyamine metabolism	18	0	0
23	nucleotide metabolism	147	6	4.1
24	biodegradation of xenobiotics	24	1	4.2
25	C1 metabolism	33	0	0
26	miscellaneous enzyme families	1219	69	5.7
27	RNA	2296	85	3.7
28	DNA	422	43	10.2
29	protein	3628	157	4.3
30	signalling	1157	81	7
31	cell	655	12	1.8
33	development	405	31	7.6
34	transport	951	32	3.4

35	35.1. not assigned. no ontology	3276	437	13.3
	35.2. not assigned. unknown	15571	31	0.2

	Σ	35246	1728	4.9

The grapevine (*Vitis vinifera*) genome sequence has recently become available [8,9] and approximately 30,000 genes were predicted. An accurate gene prediction and gene annotation is however still lacking for the whole sequence. Using strict rules for homology definition, around half of the predicted genes in the grape genome are specific for grape [9], leading to the conclusion that some of the significant metabolic pathways for grape might not be easily inferred by homology transfer. We have therefore chosen to characterize a few pathways, important for wine production and quality, in a greater detail. To this aim we constructed new pictorial representations and, where necessary, rearranged BIN representations in order to get a better overview on phenylpropanoid, terpenoid and carotenoid biosynthesis. Grape secondary metabolites, particularly polyphenols and terpenoids, have a strong influence on wine quality since they determine colour, bitterness, astringency and aroma [10,11]. They also have important pharmacological effects acting as health-promoting compounds (for a review see [12]).

Most phenolics derive from the nonoxidative deamination of the amino acid phenylalanine via phenylalanine ammonia-lyase (PAL), and encompass a range of structural classes such as lignins, phenolic acids, flavonoids and stilbenes. A key branching point in this biosynthetic pathway is the condensation of 4-coumaroyl-CoA and malonyl-CoA which can produce either trans-resveratrol (stilbene pathway), or tetrahydroxychalcone (flavonoid pathway) due to the action of stilbene synthase or chalcone synthase, respectively. Grapevine flowers and fruits are rich in flavonoids where they act as pollinator attractants and seed dispersers, UV-scavengers and are involved in disease resistance [13]. In red grape, flavanols and anthocyanins are the most abundant flavonoid classes, the latter accumulating mostly in berry skin and the former in seeds [14]. Stilbenes content increases in grapevine in response to biotic and abiotic stress [15,16], but also during berry ripening [17]. Resveratrol, the first stilbene phytoalexin identified in grapevine [18] has also been associated with the health benefits of red wine [19]. Terpenoids are a very large and diverse class of metabolites synthesized starting from isopentenyl diphosphate and dimethylallyl diphosphate via the mevalonate or the mevalonate-independent pathways. They play an important role in plant growth and development as well as in plant interaction with environment [20].

Véraison is a transitional phase of grape berry development, during which growth declines and berries start to change colour and soften. In a previously conducted study [21], three time points were selected in order to investigate fruit ripening. Time-point A (TP A, two weeks before véraison) was characterized by small green berries still accumulating organic acids, TP B (3 days before véraison) was characterized by berries in the green hard state with maximum acidic content and TP C (three weeks after véraison) by ripening berries growing fast, colouring, softening and accumulating sugars. These time points correspond to the developmental stages E-L 33, E-L 34 and E-L 36 according to the modified E-L system reported in [22]. Due to the economic importance wine has, disease research on grapevine is very important. Although grapevine fungal diseases have a major economical impact, and although they have been extensively described from a physiological standpoint, still little is known about the molecular basis of grapevine response to fungi. Among the numerous diseases affecting grapevines, eutypiosis, caused by fungus *Eutypa lata*, is very damaging. It is present in all grape growing areas around the world and causes important economic losses. After initial infection, a lag phase of several years is often observed before the appearance of symptoms whose intensity on a given plant may vary with each year. However, infected plants die within a few years. There is no known resistant cultivar, no efficient treatment and neither diagnostic tool available for this disease. Therefore a better insight into the grapevine response to *E. lata *infection is required.

While the gene model based on the genome sequence has still not been released, a large grapevine transcript database is available, including 34,134 unique sequences (*Vitis vinifera *gene index release 5, VvGI) which were used to construct several microarray platforms. The two most comprehensive ones are GeneChip^®^Vitis vinifera Genome Array from Affymetrix which has been available since 2005 and interrogates 14,496 transcripts and a ready to print 70-mer *Vitis vinifera *(grape) AROS V1.0 Oligo Set (Operon, Qiagen) covering 14,562 transcripts. Both were extensively exploited for genome-wide gene expression analyses [21,23-31]. In order to expand MapMan ontology to grapevine we have annotated the tentative gene sequences from grape gene index (*Vitis vinifera *gene index, VvGI) and have implemented them for use with Operon and Affymetrix microarrays experiments. Due to specificities of the grape physiology, we have created new pictorial representations of the grape mapping file focusing on three selected pathways: carotenoid, terpenoid and phenylpropanoid. The visualization of differentially expressed (DE) genes involved in berry development, first published in [21] is discussed as an example of application to the Affymetrix microarray platform. We have additionally performed the first analysis of processes underlying the pathogenesis of *Eutypa lata *– grapevine interaction using DNA microarrays in combination with the newly developed visualisation tool.

## Results and discussion

### Annotation of grapevine Gene Index

Annotation of grapevine Gene Index (VvGI version 5) using the MapMan ontology was performed by including information on grapevine genome and plant protein domains, the latter found in SwissProt/Uniprot plant proteins PPAP [32], the Conserved Domain Database CDD [33], Clusters of orthologous groups KOG [34] and InterPro [35]. Manual annotation was performed by different contributors for the BINs they have most expertise for. A manual correction usually consisted of blasting the appropriate tentative contig (TC) sequence, followed by classification using expert knowledge and a literature search. Altogether, 1728 manual corrections of automated annotation were made using this approach (Table 1). Because many genes from VvGI are expected to be grape-specific, special emphasis was put on genes present on either array and classified into BIN 35.2, where typically genes with no or only weak similarity to Arabidopsis and other plants included in MapMan are found. We were able to successfully annotate 13 TCs from this BIN. Some BINs, usually the smaller ones and the ones where a weaker emphasis was assigned, had a lower number/percentage of clones manually checked and corrected when necessary. Other BINs (e.g. BIN 16), which served as the basis of constructing new pictorial representations for phenylpropanoid, carotenoid and terpenoid metabolism were more thoroughly checked. A special case is BIN 20, where half of it was manually corrected due to recent changes in its annotation [7]. 34 TCs from the grape Gene Index were found to have high similarity to grapevine pathogen sequences, for example *Ralstonia solanacearum *or do not belong to *Vitis vinifera *and thus they were assigned to BIN 35.2 (not assigned. unknown). 24 out of these 34 TCs can be found in the more recent grape Gene Index (VVGI version 6).

To enable visualisation of results of transcriptomics experiments two final mapping files were created, one to be used with Operon microarrays and the other to be used with Affymetrix microarrays. Both final mapping files thus consist of the following data: BINcode, BINname, probe identifier from Operon/Affymetrix microarrays, respectively and gene description from grape gene index (with grape gene index and the protein domains information included). Due to specificities of grape physiology, we have created new pictorial representations of the grape mapping file focusing on three selected pathways: carotenoid pathway, terpenoid pathway and phenylpropanoid pathway.

### Validation of mapping file for Affymetrix platform

**Figure 1:**
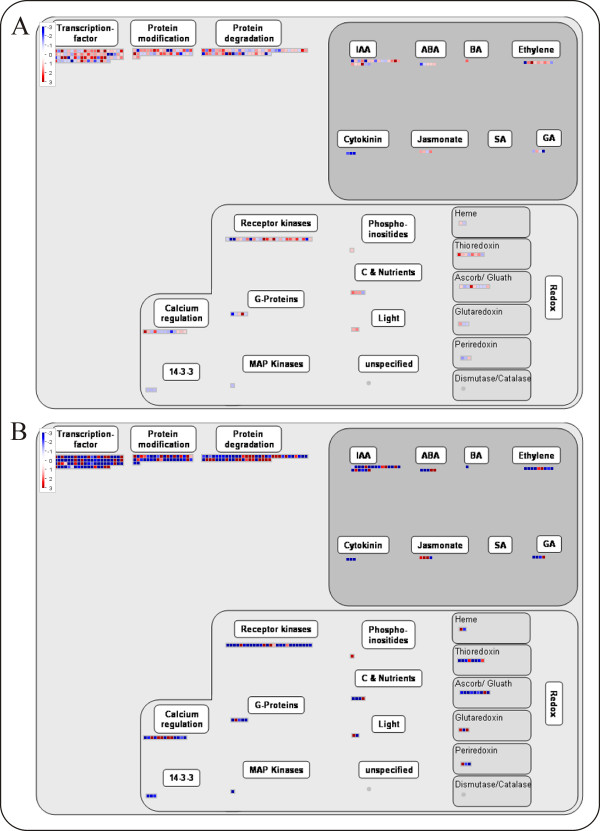
**Berry ripening gene regulation**. MapMan overview of Pinot Noir grape berry gene regulation during ripening. The modulation of the 1477 transcripts which represent the ripening core-set is shown in pair wise comparisons: time point A vs time point B (top), time point C vs time point B (bottom). The three time points correspond to three stages around véraison: 2 weeks before, 3 days before and 3 weeks after, respectively.

**Figure 2:**
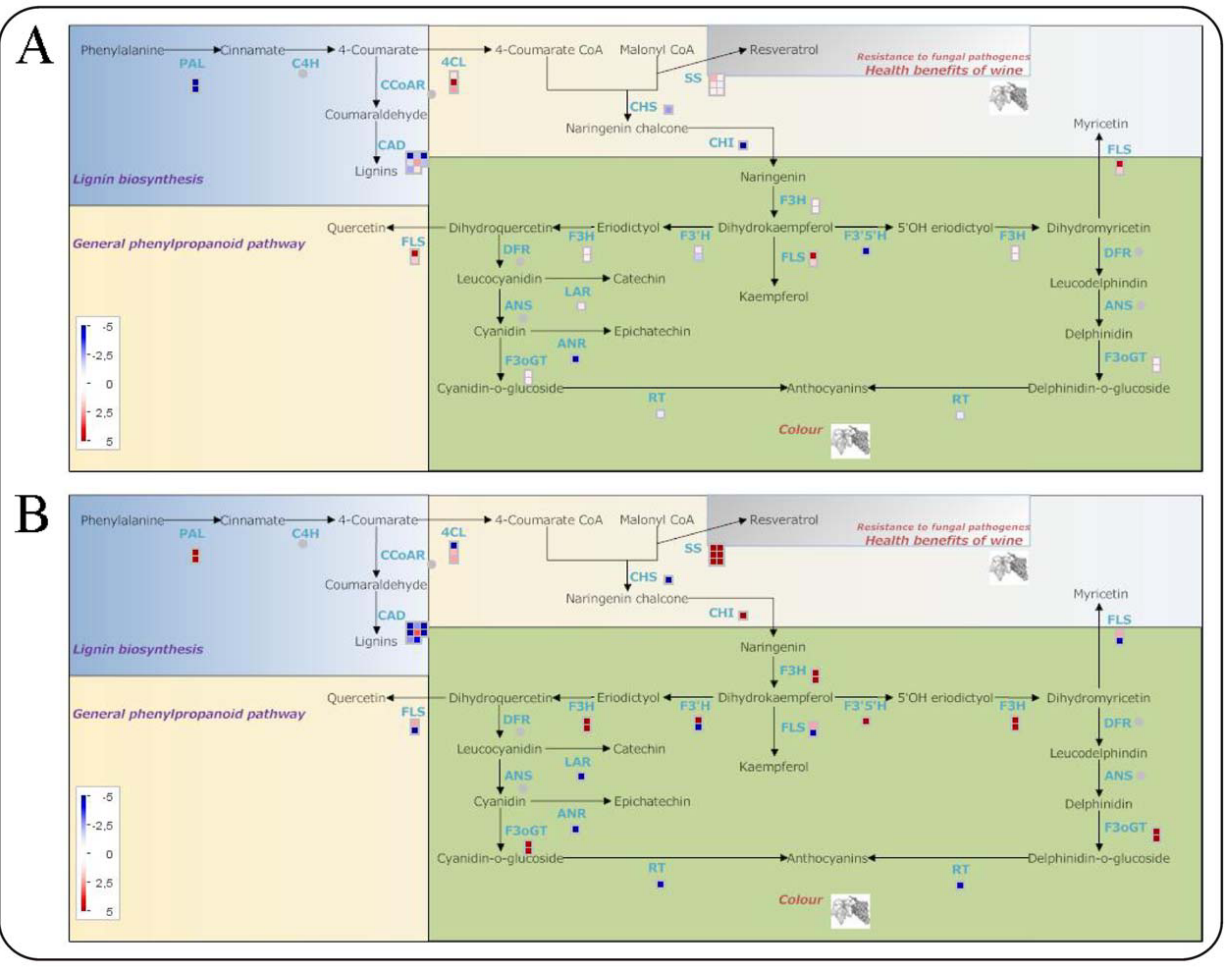
**Berry ripening phenylpropanoid pathway**. MapMan visualization of the phenylpropanoid pathway modulation during Pinot Noir grape berry ripening: time point A vs time point B (A), time point C vs time point B (B).

The mapping file built for the analysis of data obtained with the Affymetrix GeneChip was validated on a dataset of genes differentially expressed during berry ripening, reported in [21]. This study analysed two pre-véraison (A, B) and one post-véraison (C) stages during Pinot Noir grape berry development along three years, identifying a set of 1477 genes conservatively modulated. They were manually annotated using the Gene Ontology vocabulary and grouped into biological process categories. Here, we have annotated the 1477 genes with the MapMan mapping file. Two processes important for berry development have been chosen for visualization in Figure 1 and 2: an overview of ripening regulation and the phenylpropanoid pathway. The representation of berry development regulation provided by MapMan immediately highlights the large number of genes involved in the berry developmental control (226), including 95 genes involved in transcription regulation, 49 in the hormonal metabolism and 88 in signalling and protein modification (Figure 1). A clear trend of prevalent gene induction from stage A to B and prevalent repression from stage B to C is evident. These observations are in agreement with the results reported in [21], in which these three classes (transcription factors, hormone metabolism and signal transduction) included respectively 125, 65 and 92 genes. The higher numbers are probably due to a wider functional classification and to annotation redundancy of the published analysis. The general trend of these classes is the same as reported in Figure 5 of [21], in which it is evident that all of them are prevalently induced from stage A to B and then mostly repressed. The main advantage of using MapMan is no doubt the ease and speed of the analysis.

The visualization of the genes distribution among the phytohormones confirms the large involvement of genes linked to auxins and ethylene and fewer genes involved in the abscisic acid, brassinosteroid and gibberellic acid pathways as previously reported [36-38], and as showed in Table 1 of [21].

Finally, the two categories of receptor kinases and calcium regulation (Figure 1), which were not investigated in detail in the published analysis [21], appear to be quite highly represented according to the MapMan annotation and, in agreement with [25]. Nonetheless, the two categories of light signalling and redox control, on which the published analysis had focused (see Table 1 and Figure 7 in [25]), include fewer transcripts and gene families, respectively.

This comparison shows that MapMan is a reliable annotation and data display tool which is easy to use and particularly efficient for representing genome-wide gene expression experiments. Obviously, when the interest is focused on a particular pathway or metabolism, further investigation is needed.

The pictorial representation of the general phenylpropanoid pathway is very effective, as it displays the information about the members of the gene families, the relative gene expression and the name of the enzyme in a single image. It is interesting to observe that before véraison the pathway is not modulated, with the exception of the flavonol synthase gene which is responsible for the flavonols biosynthesis. On the contrary during ripening, specific enzymes are strongly induced: phenylalanine ammonialyase (PAL), at the beginning of the pathway; stilbene synthase (SS), responsible for resveratrol accumulation; chalcone isomerase (CHI) and flavanone-3 hydroxylase (F3H), at the beginning of flavonoid synthesis; UDPglucose:flavonoid 3-O-glucosyltransferase (F3OGT), catalysing the final steps of colour accumulation. The picture clearly shows that, at the same time, the genes encoding the enzymes leucoanthocyanidin reductase (LAR) and anthocyanidin reductase (ANR) are repressed. Altogether these results suggest that in the ripening process the branches of the phenylpropanoid pathway which lead to the accumulation of stilbenes and anthocyanins are favoured with respect to those which lead to tannins production.

### Overview of transcriptional changes in grapevine response to infection with Eutypa lata

**Figure 3:**
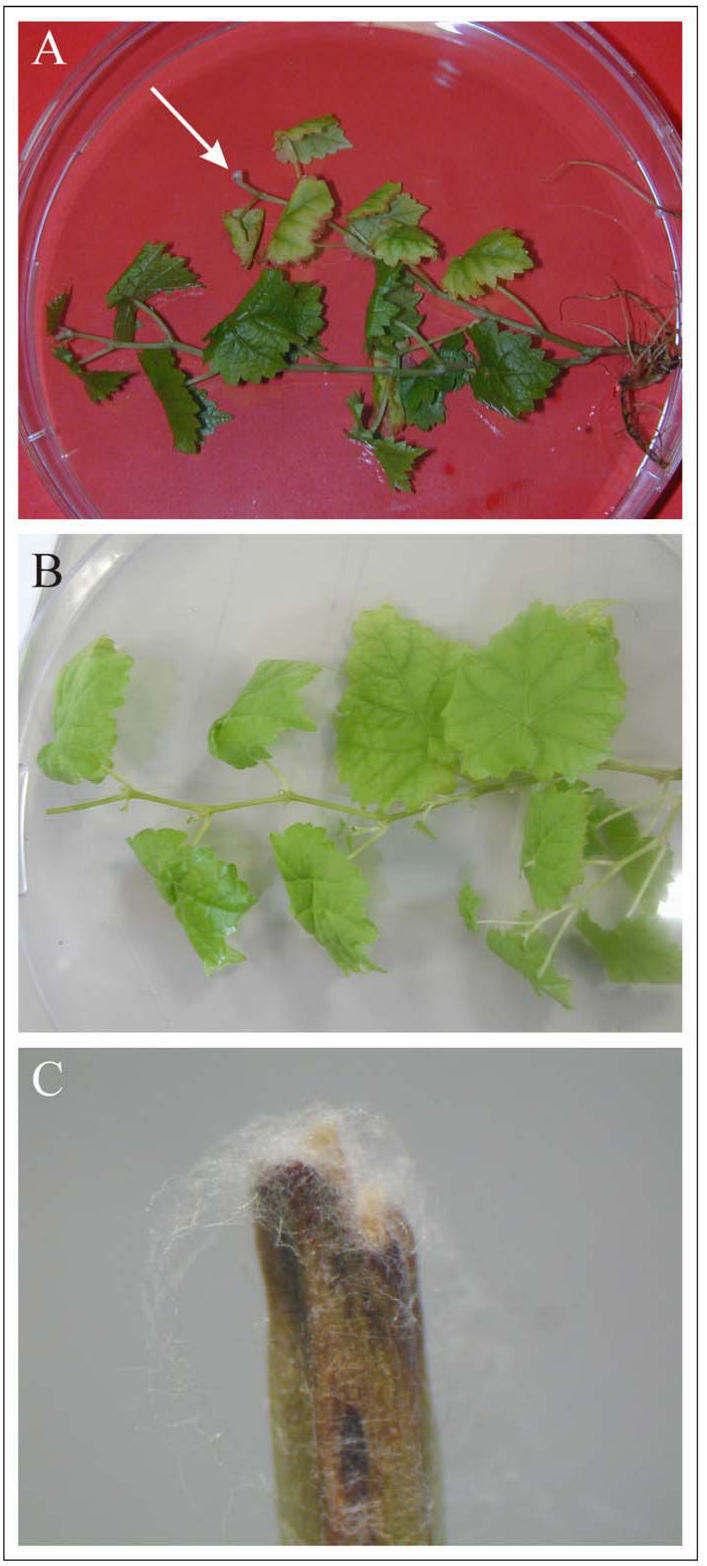
**Eutypiosis symptoms**. Eutypiosis symptoms on grapevine plantlets (A) as compared to control plants (B) 7 weeks after infection. Arrow indicates the point of infection to the infected cut stem which is shown in a close up on (C) to see typical necrosis caused by the disease.

20 Cabernet Sauvignon plantlets were experimentally infected with NE85-1 *E. lata *strain in three independent series. Seven weeks after infection, the plantlets with confirmed eutypiosis and healthy plantlets were collected for the microarray hybridisations each in two technical replicates. The symptoms consisted of a clear necrosis appearing few mm above the infected zone (Figure 3C). Symptoms sometimes extended beyond this zone, inducing leaf yellowing and necrosis (Figure 3A). Similar symptoms were obtained after infection of various grapevine varieties with either *E. lata *mycelium (symptoms observed after 6 weeks) or culture filtrate (symptoms observed after 4 weeks) [39].

Altogether 312 differentially expressed (DE) genes were identified using strict statistical testing (Bonferroni adjusted p < 0.05, see Additional file 1: Differentially expressed genes in Eutypa lata experiment). We have however used less stringent statistical testing to get an pictorial overview of metabolic and signal processes involved using the expanded MapMan tool (non-adjusted p < 0.01; 767 differentially expressed genes). DE genes were mapped into 29 out of 34 BINs represented in MapMan and the ones most significantly altered are shown in Table 2. Interestingly, except few individual enzymes (invertase TC67908, beta-galactosidase TC53602 and GDSL-motif lipase TC59023) that were significantly down regulated, all pathways represented by DE genes were found to be up-regulated. However, 302 DE genes (39%) did not have a reliable annotation and were not assigned to any pathway/process (BIN 35). Observed changes in expression were however relatively small as was the percentage of DE genes obtained. One has to note that expression changes were monitored late after infection (7 weeks after inoculation). When studying response of grapevine to powdery mildew over time, the strongest response was observed up to 24 h post inoculation, thus a stronger response could also be expected in our experimental system at shorter times post inoculation [28].

**Table 2:** Significantly altered processes

**BIN**	**Upregulated processes/protein families**	**Number of DE genes**	**Total number of genes in BIN**	**p-value**
10.5	cell wall.cell wall proteins	8	54	0.006
10.5.3	cell wall.cell wall proteins.LRR	4	35	0.006

16	secondary metabolism	44	190	< 0.0001
16.1	secondary metabolism.isoprenoids	9	57	0.008
16.2	secondary metabolism.phenylpropanoids	15	45	< 0.0001
16.2.1	secondary metabolism.phenylpropanoids.lignin biosynthesis	14	31	< 0.0001
16.8	secondary metabolism.flavonoids	18	55	< 0.0001
16.8.2	secondary metabolism.flavonoids.chalcones	5	9	0.0009
16.8.3	secondary metabolism.flavonoids.dihydroflavonols	6	16	0.004

17.5.1	hormone metabolism.ethylene.synthesis-degradation	6	32	0.01

20	stress	49	396	0.001
20.1	stress.biotic	26	157	< 0.0001
20.1.7	stress.biotic.PR-proteins	20	64	< 0.0001

26.4	misc.beta 1,3 glucan hydrolases	3	19	0.005

27	RNA	57	970	0.01
27.3	RNA.regulation of transcription	53	737	0.01

29.5.15	protein.degradation.inhibitors	4	14	0.007

Significantly altered processes or protein families according to changes in gene expression level in symptomatic compared to healthy grapevine leaf samples. The results show numbers of genes annotated to each process or family (indent) and corresponding p-values (as calculated by MapMan Wilcoxon rank sum test) according to the MapMan gene ontology.

### Stress related responses in E. lata infected plants

**Figure 4:**
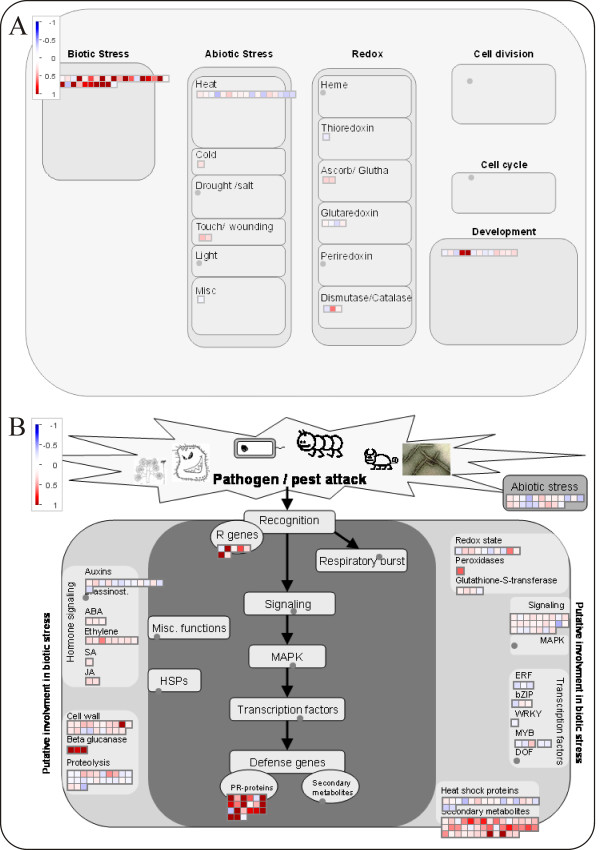
**Eutypa infection response**. Overview of cellular responses in grapevine leaves to Eutypa infection (A) and more specifically responses of genes related to biotic stress (B) as visualised by MapMan. Genes that were shown to be differentially expressed using p < 0.01 as a cutoff value were imported.

In Figure 4A we can see that changes in expression of genes involved in cellular responses clearly indicate that the only genes induced belong to the category of biotic stress. The exceptions are the two genes classified to development corresponding to legumins, a gene family that has been frequently mentioned as up-regulated by biotic stress [40]. This is more specifically shown in Figure 4B showing biotic stress related genes which 107 out of the 312 strictly defined DE genes are annotated to. The strongly regulated biotic stress related genes include several PR-proteins (endochitinase (TC60929), chitinase (Q7XB39), osmotin-like proteins (P93621), thaumatin (Q7XAU7), disease response protein (Q45W75), tumor related proteins (P93378)), three 1,3-*β*-glucanases and several proteinase inhibitors. Early induction of genes encoding chitinases and 1,3-*β*-glucanases is a typical response of plants towards fungal pathogens. In the interaction between *Cladosporium fulvum *and tomato, resistance against the fungus correlates with early induction of transcription of genes encoding apoplastic chitinase and 1,3-*β*-glucanase and the accumulation of these proteins in inoculated tomato leaves [41]. It is additionally considered that genes encoding chitinases or *β*-1,3-glucanases are the most attractive candidates for the genetic manipulation approach to increase anti-fungal tolerance in grapevine [42]. Also strong induction of polygalacturonase inhibiting proteins (PGIPs, TC69081 CF604851 TC69081) was observed. In plant tissues, the activity of pathogen's PGs is counteracted by PGIPs, leucine-rich repeat proteins (LRR) located in the cell wall. A role for PGIPs in plant defence has been demonstrated by showing that transgenic *Arabidopsis *plants over-expressing PGIPs exhibit enhanced resistance to *Botrytis cinerea *[43].

### Induction of phenylpropanoid pathway in eutypiosis

**Figure 5:**
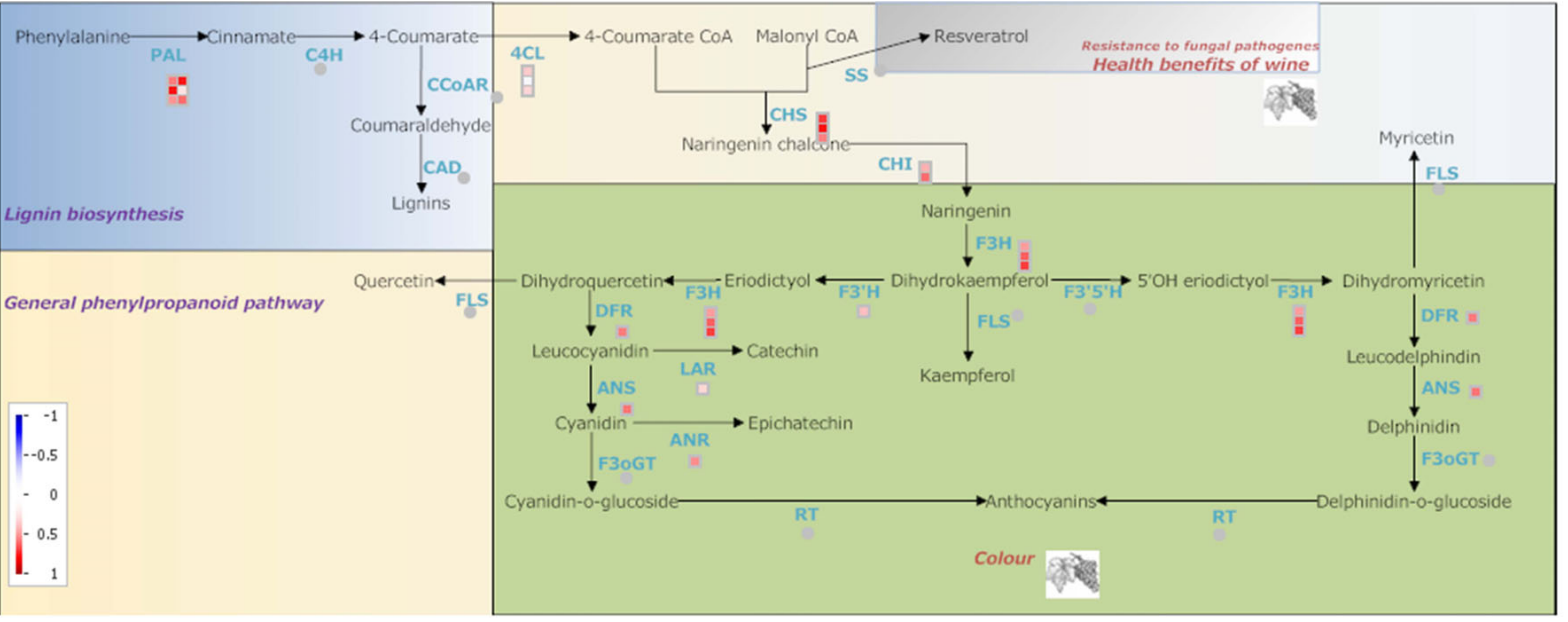
**Eutypa infection phenylpropanoid pathway**. Changes in expression of genes involved in phenylpropanoid metabolism after *E. lata *infection.

Secondary metabolism was also strongly induced after infection of grapevine by *E. lata *(Figure 5). Phenylalanine ammonia-lyase, PAL (P45735 and P45726), the first enzyme of the pathway, was up-regulated 1.5 to 1.7 fold. Likewise, chalcone synthase (Q8W3P6, O80407), chalcone isomerase (P51117), dihydroflavonol 4-reductase, DFR (P93799), and anthocyanidin reductase, ANR (Q7PCC4) which control the pathways leading to flavonols, tannins, and anthocyanins were up-regulated. Also the induction of secondary metabolites was shown to be associated with defence and pathogenesis related processes [44].

However, these results are not in complete agreement with those obtained by treating grapevine cell suspension cultures with the *Eutypa *pathogenic toxin, eutypine. In that experiment the authors observed a strong repression of the UDP glucose-flavonoid glucosyltransferase gene (UFGT) and no significant modulation of the chalcone synthase and DFR encoding genes [45]. A possible explanation of the incongruity can be different experimental systems used: complex response to the pathogen is most probably going to be different in a population of relatively uniform cells of a cell culture than in whole plants. In addition, the treatment with toxic compounds alone might not be directly related to effects of the real infection with pathogen.

Similarly, no changes in activity of respiratory metabolism related genes were observed in infected plantlets, while a model bioassay using the yeast *Saccharomyces cerevisiae *showed that the *E. lata *derived toxic metabolites inhibited the respiratory metabolism, causing the reduced growth of the cells. One possible explanation is that such a response of grapevine cells is masked due to the dilution of infected area with surrounding healthy tissue. The other explanation is that grapevine reacts differently from yeast and that primary target of toxic compounds is not yet determined [46].

## Conclusion

Biological interpretation of data is the last step in microarray-based analysis of transcriptome. Several tools that enable easier interpretation of plant microarray data are available (e.g. [47,48]), but have usually been designed specifically for *Arabidopsis *microarrays. On the other hand MapMan has an advantage in its flexibility, as it can be extended easily to any plant species. Here, we present the extension of MapMan ontology to grapevine experiments, performed with Affymetrix or Operon microarrays. Validation of Affymetrix mapping file was performed through biological re-interpretation of previously published grapevine berries development gene expression data [21]. Together with the construction of pictorial representations for carotenoid, terpenoid and phenylpropanoid metabolism, a deeper insight into changes in these metabolic activities was possible. Recently, grapevine genome has become available [8,9] and complete gene annotations are yet to be done. Improved MapMan grapevine annotation will be an ongoing process through expert manual editing and can be easily modified when the genome annotations will become available as well as when new microarray platforms are constructed.

Data on molecular basis of grapevine defence against fungal pathogens are gradually becoming available. Powdery mildew induces defence-oriented reprogramming of the transcriptome in a susceptible but not in a resistant grapevine [28]. In addition, possible innate resistance against pathogenic fungi is being unravelled using transcriptional and metabolic profiling of grape (*Vitis vinifera *L.) leaves [49]. Here we present an overview of the transcriptional changes in a susceptible grapevine – *Eutypa lata *interaction at the time of symptoms development. The results obtained through the use of the MapMan representation of metabolic pathways showed that the responsive genes belong to families known to be involved in the plant defence towards fungal infection (PR-proteins, enzymes of the phenylpropanoid pathway). Nevertheless, the observed response was relatively weak. This is in line with the hypothesis that defence compounds are induced in resistant as well in susceptible interaction, the main difference being the speed and intensity of response. Also, it should be stressed that not all responses to pathogens necessarily occur at transcriptional level, and that translational and post-translational events may also be involved.

## Methods

### Plant material

Cabernet Sauvignon *in vitro *plantlets were experimentally infected with the *E. lata *strain NE85-1 previously characterized as an aggressive strain [50]. Cabernet Sauvignon is a major variety used in various countries around the world and is particularly sensitive to *E. lata*. Infection was achieved by applying 10–15 days growing mycelium directly onto the cut surface of de-topped plantlets. Uninfected de-topped plantlets were used as healthy controls. Samples were further on characterized according to symptoms, re-isolation of the fungus, and formal identification of re-isolated *E. lata *mycelium by PCR. Foliar symptoms of eutypiosis were evaluated for each plantlet seven weeks after the experimental infection. 46 plantlets out of 60 infected plantlets were showing eutypiosis symptoms. The control plantlets [40] did not show any symptoms. After inspection of symptoms, each plantlet was frozen individually in liquid nitrogen and stored at -80 ‰.

### Confirmation of infection

*E. lata *was re-isolated from the first internode of each plantlet. Internodes were surface-sterilized for 2 min with 1% sodium hypochlorite, rinsed in sterile water three times for 5 min, cut into two pieces and put on sterile PDA medium containing streptomycin (0.1 mg mL^-1^). After incubation in darkness for 10 days at 22 ‰ the plates were visually assessed for the presence of typical *E. lata *cottony white mycelium. DNA was extracted from mycelium and *E. lata *was detected with PCR as described in [51]. The re-isolation and PCR detection of *E. lata *was used to check whether the non inoculated control was indeed axenic and to distinguish the experimentally inoculated plantlets that became infected from those that did not. Fungus was successfully re-isolated from all plantlets that were characterised as symptomatic and its identity confirmed by PCR. From the non-symptomatic plantlets no fungus could be isolated. The plantlets where infection was not successful were eliminated from further analysis.

### Microarray hybridizations

RNA was isolated as described in [52]. To prepare fluorescently (cy3/Cy5) labelled antisense RNA (aRNA) targets, total RNA was amplified and purified using the Amino Allyl MessageAmp II aRNA Amplification Kit (Ambion, USA) following the manufacturer's instructions.

Array-Ready Oligo Set™ for the Grape (*Vitis vinifera*) Genome Version 1.0 designed and synthesised by Operon (Qiagen) was used for microarrays fabrication. Probes were spotted on epoxy mirror slides (Amersham, GE HealthCare) at the Montpellier Languedoc Roussillon Genopole, Institut de Génomique fonctionnelle. Just before hybridization, oligonucleotides were fixed on the slide by UV (254 nm) radiation of 120 mJ in a UV Stratalinker 2400-crosslinker (Stratagene). The slides were then washed twice in 0.2% SDS and positioned into the hybridization chambers. Approximately 4 *μ*g of Cy3 and 4 *μ*g of Cy5-labelled aRNA was mixed, fragmented, volume brought to 100 *μ*L with hybridization buffer and denatured.

Hybridization was then conduced 16 h at 37 ‰ with medium agitation in the automated microarray station HS4800 Mastersystem (Tecan). Slides were washed sequentially with 1× SSC/0.2% SDS for 20 min, twice with 0.1× SSC/0.2% SDS for 10 min and finally with 0.1× SSC for 10 min. Washed arrays were quickly dried with nitrogen gas (2.7 × 10^5 ^Pa) and immediately scanned with Genepix 4000B fluorescence reader (Axon Instruments, Canada) using GenePix 4.0 image acquisition software.

### Statistical analysis of microarray results

Signals were extracted from microarray images using the Maia tool version 2.75 [53]. Median intensity gene expression data without background subtraction were normalized by a global lowess method followed by a print-tip median method with a modified version of the Goulphar script version 1.1.2 [54]. Differentially expressed genes were identified with the R/Bioconductor package Limma[55] using linear models and by taking into account technical and biological replicates.

### Microarray data accession number

Microarray data analyzed in this study have been deposited in the ArrayExpress database. The accession number is E-MEXP-2102 [56].

### Classification of TIGR Gene Index using the MapMan ontology

*Vitis vinifera *Gene Index sequences were downloaded from TIGR (VvGI version 5; 34,134 unique sequences). These were blasted (BLASTx, version 2.2.14) against Arabidopsis proteins release TAIR7 [57] under default settings. A RPS-blast against SwissProt/Uniprot plant proteins PPAP [32], the Conserved Domain Database CDD [33], Clusters of orthologous groups KOG [34] and InterProScan [35] was also performed.

The results of all searches were compiled into one table and using the Arabidopsis as well as RPS-Blast hits an initial classification into the MapMan BINs was achieved. This was then checked manually based on the annotations provided by TIGR, as well as based on the results obtained by the different similarity searches [7]. Furthermore several genes were annotated based on the expertise of the different contributors. The final mapping file has 35,246 entries, of those 34,132 are unique and the others are represented in two or more subBINs.

### Grapevine specific BINs and subBINs organization

In order to get pictorial representations for the selected pathways (carotenoid, terpenoid and phenylpropanoid) BIN structures for the secondary metabolism had to be further refined. The carotenoid biosynthesis pathway was adopted from potato carotenoid pathway [58] with the help of already published MapMan representation for potato [7]. The flavonoid biosynthesis pathway was constructed based on [24,59] where grape gene names, playing a role in flavonoid biosynthesis were directly used for creating our improved, grape-based flavonoid pathway. The existing pathway mapping file for terpenoid biosynthesis from the MapMan website [60] which included only the mevalonate pathway was modified in order to include the non-mevalonate pathway, too. Thus the present terpenoid mapping file includes both the mevalonate pathway and non-mevalonate pathways, which are essential for plant development [16]. Pictorial representations for all three pathways can be uploaded from the MapMan website.

### Linking the mapping file to Operon and Affymetrix microarrays

In order to make the mapping file useful for studies involving grape microarray experiments, information from two microarray platforms was used, the Array Ready Oligo Set *Vitis vinifera *(grape) AROS V1.0 Oligo Set (Operon, Qiagen) covering 14,562 transcripts and the GeneChip^® ^*Vitis vinifera *Genome Array with 14,496 probesets (Affymetrix). Sequences from both microarrays were blasted against the grape gene index and grapevine genome sequences using the default parameters (BLASTN [61]). Since the first hit from this blast was typically by orders of magnitude (as shown with the E value) better than the other hits, only the information from the first hit was taken into account. Hits displaying less than 70% sequence identity were ignored. Subsequently all found blast hits were aligned to the corresponding oligonucleotide using the smith-waterman algorithm (as implemented in the "water" program of the EMBOSS package [62]), again discarding hits with more than 10% gaps and/or less than 70% sequence identity over the full length of the oligonucleotide sequence. The remaining hits were classified into MapMan BINs [63] using the manually curated mapping file for grapevine as a reference. If all hits for one specific oligonucleotide probe belonged to the same BIN (or multiple BINs) these BINs were assigned to the oligonucleotide probe. In case of disagreeing hits the BIN code "35.3 not assigned. disagreeing hits" was assigned unless manual inspection suggested a different BIN. For each oligonucleotide that showed multiple hits, the corresponding gene identifiers of all found hits were given in the description line in order not to lose potentially important information.

### Berry development study

Details on experimental setup, lab work and statistical analysis of microarray results are described in [21]. Briefly, 50 berries of *Vitis vinifera *cv. Pinot Noir were randomly chosen from ten grape clusters harvested at the peak of acidity, two weeks before and three weeks after in the 2003, 2005 and 2006 seasons. Data representing individual years (2003, 2005 and 2006) were analyzed independently in order to obtain one set of differentially expressed genes for each season. Following stringent data analysis, a set of 1477 genes modulated during berry ripening was obtained. Genes were grouped into 17 functional categories based on GO 'biological process' terms annotation.

## Authors' contributions

AR, BU, ML and MS constructed an initial automated mapping file. CK and ML were involved in the mapping of TCs to probe(sets). SP, CM, AR, MH, BU were involved in manual annotation of TCs. CC, SD and PCT were involved in design setup and analysis of Eutypa lata experiment. SP and CM were involved in validation grapevine MapMan for Affymterix microarrays. KG was involved in organization of the work and writing of the manuscript.

## Supplementary Material

Additional file 1 **ID, VVGI5id, VVGI5annotation, UniqueOligo, logFC, AveExpr, t, P.value, adj.P.value, B**. The data shows all 312 differentially expressed genes as found in the *E. lata *experiment: their microarray ID, VVGI5 ID and its annotation, unique oligo sequence, M and A values, p-value and Bonferroni-adjusted p-value, t-statistic and B-statistic. Click here for file
